# Violence directed at healthcare staff by children and adolescents treated in mental health wards

**DOI:** 10.1192/j.eurpsy.2026.10571

**Published:** 2026-07-31

**Authors:** N. Manevich Lozovsky, I. Bluvstein, M. Itzhaki

**Affiliations:** 1 The Department of Nursing Sciences, Tel Aviv University, Tel Aviv; 2 Merhavim Medical for Brain and Mind Treatment, Be’er Ya’akov - Ness Ziona, Israel

## Abstract

**Introduction:**

Violence in mental health hospitals has been on the rise, yet little research has focused on violence within child and adolescent mental health wards. Such exposure is associated with increased stress and occupational strain, negatively impacting the professional performance and the perceived safety of caregivers.

**Objectives:**

This study explored the impact of child and adolescent violence on healthcare staff in mental health wards, focusing on its relationship with emotional labor, PTSD symptoms, and physical and mental fatigue.

**Methods:**

A cross-sectional design was employed with 113 healthcare professionals from a large mental health hospital, including physicians, nurses, social workers, occupational therapists, and psychologists. Data were collected using a five-part questionnaire assessing self-reported exposure to violence, post-traumatic stress disorder (PTSD) symptoms, emotional labor, multidimensional fatigue, and demographic variables. Hierarchical linear regression and moderation analyses were conducted using PROCESS software (v.4).

**Results:**

Participants reported high levels of verbal (93.8%) and physical (74.3%) violence from children and adolescents under their care. No direct association was found between violence and emotional labor, possibly reflecting staff normalization of violent behavior due to the complex backgrounds of their patients. Significant correlations were found between PTSD symptoms and both verbal (r = .37, p < .001) and physical (r = .50, p < .001) violence, as well as between emotional labor and physical/mental fatigue (r = .27, p = .004). PTSD symptoms moderated the relationship between exposure to violence and fatigue (β = .21, CI: 0.11–0.32).

**Conclusions:**

While emotional labor assists staff in regulating emotions, it also contributes to fatigue, particularly in the context of trauma. Interventions should focus on reducing violence in child and adolescent mental health wards and increasing awareness of post-traumatic symptoms among healthcare professionals.

**Disclosure of Interest:**

None Declared

